# Shaping Nanoparticles for Interface Catalysis: Concave Hollow Spheres via Deflation–Inflation Asymmetric Growth

**DOI:** 10.1002/advs.202000393

**Published:** 2020-05-19

**Authors:** Rongtai Yu, Xiaodan Huang, Yang Liu, Yueqi Kong, Zhengying Gu, Yang Yang, Yue Wang, Wenhuang Ban, Hao Song, Chengzhong Yu

**Affiliations:** ^1^ School of Materials Science and Engineering Jingdezhen Ceramic Institute Jingdezhen Jiangxi 333403 P. R. China; ^2^ Australian Institute for Bioengineering and Nanotechnology The University of Queensland Brisbane Queensland 4072 Australia; ^3^ School of Chemistry and Molecular Engineering East China Normal University Shanghai 200241 P. R. China

**Keywords:** hollow nanospheres, interface catalysis, nanostructures, pickering emulsion

## Abstract

Hollow spheres are charming objects in nature. In this work, an unexpected deflation–inflation asymmetric growth (DIAG) strategy is reported, generating hollow nanoparticles with tailored concave geometry for interface catalysis. Starting from aminophenol‐formaldehyde (APF) nanospheres where the interior crosslinking degree is low, fully deflated nanobowls are obtained after etching by acetone. Due to APF etching and repolymerization reactions occuring asymmetrically within a single particle, an autonomous inflation process is observed similar to a deflated basketball that inflates back to a “normal” ball, which is rare at the nanoscale. A nucleophilic addition reaction between acetone and APF is elucidated to explain the chemistry origin of the DIAG process. Interestingly, the deflated APF hollow spheres enable preferential immobilization of lipase in the concave domain, which facilitates the stabilization of Pickering emulsion droplets for enhanced enzymatic catalysis at the oil–water interface. The study provides new understandings in the designable synthesis of hollow nanoparticles and paves the way toward a wide range of applications of asymmetric architectures.

## Graphical abstract

Concave hollow nanospheres are tailored through an unprecedented deflation–inflation asymmetric growth strategy, with both the structural evolution and chemistry origin of this process clearly elucidated. The unique concave architecture favors localized enzyme immobilization, enabling oriented assembly at the oil–water interface of Pickering emulsion stabilization for enhanced enzymatic catalysis.

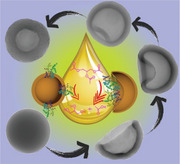

## 1 Introduction

Enormous objects in nature appear as hollow, from bubbles to cells, in some cases featuring an elastic surface which brings in a variety of intriguing structures and functions.^[^ ^1^, ^2^ ^]^ Inspired by nature, extensive efforts have been made to construct artificial hollow nanostructures with spherical to polyhedral morphologies^[^ ^3^, ^4^ ^]^ or single to multishelled architectures.^[^ ^5^, ^6^ ^]^ To realize a hollow interior, various methods have been developed, including soft/hard‐templating,^[^ ^7^, ^8^, ^9^ ^]^ selective etching,^[^ ^10^, ^11^, ^12^ ^]^ Ostwald ripening,^[^ ^13^ ^]^ and thermally induced mass relocation.^[^ ^14^ ^]^ These strategies typically generate nanoparticles in a symmetric configuration. Compared to symmetric nanoparticles, the heterogeneity in either structure or composition within a single particle brings tremendous unique properties.^[^ ^15^, ^16^, ^17^, ^18^, ^19^ ^]^ For example, asymmetric nanoparticles with a convex geometry, such as dumbbell^[^ ^20^ ^]^ or head–tail^[^ ^21^ ^]^ morphologies, were reported to stabilize Pickering emulsions and enhance interface catalysis when loaded with noble metal catalysts. However, there are rare reports using concave nanoparticles in the formation of Pickering emulsion at the biphasic interface.

Recently, particular interests have been paid to tailor hollow particles with an asymmetric geometry, such as nanobowls^[^ ^22^, ^23^, ^24^, ^25^ ^]^ and nanoflasks,^[^ ^26^, ^27^ ^]^ showing fascinating properties for photonic sensor,^[^ ^28^ ^]^ energy storage,^[^ ^22^, ^26^ ^]^ and drug delivery.^[^ ^23^ ^]^ An intact hollow sphere can be considered as a basketball with a symmetric spherical morphology, while the nanobowl can be considered as a deflated basketball. Nanoparticles with a bowl‐like geometry has been reported through the deflation of flexible hollow spheres.^[^ ^29^, ^30^ ^]^ However, unlike in daily life a deflated basketball can be inflated back to a concave or even “normal” basketball, such a process has not been reported in the nanoworld for hollow structures

**Scheme 1:**
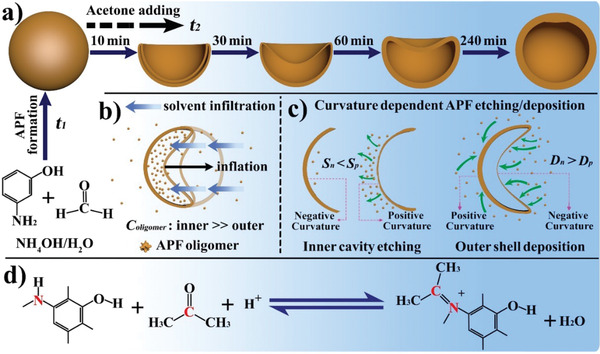
Illustration of the DIAG process and mechanism. a) The two‐step synthesis and morphology evolution of APF nanospheres. b) The inflation of a deflated nanosphere is driven by osmosis. c) The asymmetric growth is initiated by curvature dependent APF etching and deposition at inner cavity and outer shell, respectively. d) The reaction between APF with low polymerization degree and acetone in the DIAG process.

Herein, we present an unprecedented finding that hollow polymer spheres with tunable concave nanostructures can be created via a deflation–inflation asymmetric growth (DIAG) process (**Scheme** **1**a). For the first time, we demonstrate that these concave hollow particles load enzyme molecules favorably at the deflated side, further stabilizing Pickering emulsion with most enzyme exposed to the oil phase for enhanced interface catalysis. In specific, aminophenol‐formaldehyde (APF) nanospheres are formed after a given period of polymerization time (*t*_1_). Then, acetone is added to the reaction solution for another period of time (*t*_2_), initiating the DIAG process. The structure evolution originates from the heterogeneity of APF nanospheres, where the interior has a lower crosslinking degree than the outer region, thus can be etched by acetone.^[^ ^12^ ^]^ Therefore, deflated hollow nanospheres are formed similar to previous reports.^[^ ^22^, ^23^, ^31^ ^]^ Interestingly, the deflated spheres can be inflated autonomously, driven by the osmosis where APF oligomer concentration (*C*_oligomer_) is higher inner than outer cavity (Scheme 1b). The DIAG process also features an asymmetric shell growth initiated by curvature dependent APF etching and deposition (Scheme 1c). At inner cavity where APF polymer can be etched, the oligomer solubility (*S*_p_) of surface with a positive curvature is higher than that (*S*_n_) with a negative curvature, initially leading to a thinner invaginated shell compared to the intact side. At outer shell where the APF is more condensed and thus hardly etched, the APF oligomer deposition rate (*D*_n_) at negative curvature surface surpasses that (*D*_p_) at positive curvature surface. The oligomer deposition dominates over time, eventually the difference in wall thickness is minimized. Different from literature reports where acetone was regarded as a solvent,^[^ ^12^ ^]^ nucleophilic addition of acetone with amino/imino groups in low crosslinked APF polymer is revealed by capturing the metastable ─C═N species released from the etching process (Scheme 1d).

## 2 Results and Discussion

### 2.1 Deflation–Inflation Asymmetric Growth

**Figure 1:**
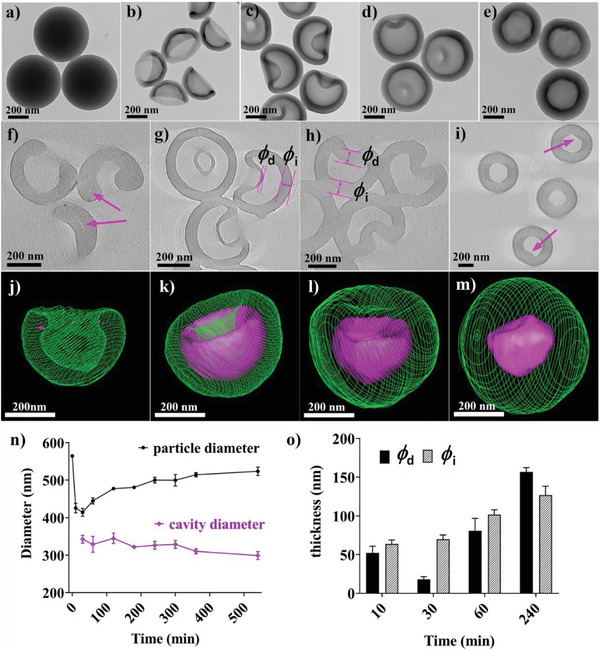
Characterization of the DIAG process. a–e) TEM images of $S_{\left(20,\;\;t_{2}\right)}$, where *t*_2_ is 0, 10, 30, 60, and 240 min, respectively. f–i) ET slices and j–m) reconstructed hollow structures of S_(20, 10)_, S_(20, 30)_, S_(20, 60)_, and S_(20, 240)_, respectively. In (j)–(m), green contours represent the outer margin of the nanoparticle while the purple region reveals inner hollow cavity. n) The mean particle and cavity diameter and o) thickness of intact/deflated shells as a function of *t*_2_.

Solid APF nanospheres were prepared in an ammonia aqueous solution, according to a literature method.^[^ ^29^ ^]^ The samples prepared after polymerization and acetone etching are named as $S_{\left(t_{1},\;\;t_{2}\right)}$. The synthesis details can be found in the Supporting Information. When the APF polymerization time (*t*_1_) was 20 min without adding acetone, transmission electron microscopy (TEM) image of S_(20, 0)_ shows a uniform particle size of ≈560 nm (**Figure** **1**a; Figure S1a, Supporting Information). After adding 30 mL of acetone into the reaction solution, samples were collected at different etching time (*t*_2_ = 10, 30, 60, and 240 min) and their TEM images are shown in Figure 1b–e, respectively. Bowl‐like nanoparticles are observed for S_(20, 10)_ (Figure 1b). Due to the fast polymerization of APF, the polymer crosslinking degree inside the sphere is lower than that in the shell.^[^ ^12^ ^]^ The components inside were easily etched by acetone within 1 min, leading to hollow spheres with a deformable shell (Figure S2a, Supporting Information). These hollow spheres were than partially deflated at *t*_2_ = 5 min (Figure S2b, Supporting Information) and then fully deflated as nanobowls at *t*_2_ = 10 min (Figure 1b), possibly due to the loss of a significant mass of solid core causing the collapse of the thin and flexible polymer shell. Moreover, the osmotic pressure drives the infiltration of solvent into the hollow cavity, which could contribute to pressing the polymer shell inward and converting hollow spheres into nanobowls.

Surprisingly, the deflated surface of S_(20, 30)_ bounced back into concave hollow spheres (Figure 1c; Figure S1c–j, Supporting Information). Increasing *t*_2_ to 60 and 240 min led to further inflated concave hollow spheres of S_(20, 60)_ with a shallow concavity (Figure 1d) and almost fully inflated symmetric hollow spheres of S_(20, 240)_ (Figure 1e). Hollow nanospheres of S_(20, 30)_ fabricated without stirring and centrifugation showed a similar concave morphology (Figure S3, Supporting Information) to the nanoparticles synthesized under vigorous stirring and high‐speed centrifugation (Figure 1c), indicating that the deflation and inflation process was not influence by the reaction parameters of stirring and centrifugation.

To reveal the detailed intricate structural features during the inflation process, a unique characterization tool of electron tomography (ET) was used.^[^ ^32^ ^]^ An ET slice of S_(20, 10)_ at varied orientations is shown in Figure 1f. Tinny void spaces can be identified indicated by red arrows, suggesting that these nanobowls (Figure 1b) are formed from almost complete deflation of hollow spheres. Such fine structural features cannot be observed from direct TEM observations. Tomogram sliced parallel or vertical to the deflated plane of S_(20, 30)_ presents double‐shell or crescent shapes (Figure 1g). The deflated shell thickness (*ϕ*_d_) is thinner than the opposite intact shell thickness (*ϕ*_i_), different from S_(20, 10)_. However, when the reaction time is 1 h, S_(20, 60)_ showed similar shell thickness at both sides (Figure 1h), similar to S_(20, 10)_. Finally, the deflated nanoparticles inflate back to almost symmetric hollow spheres after 4 h of reaction (Figure 1i). Interestingly, there still exists imperceptible curved features inside the hollow cavity of S_(20, 240)_ (indicated by arrows), indicating the inflation journey of this “normal” hollow sphere through a deflated particle.

The 3D configurations of typical $S_{\left(20,\;\;t_{2}\right)}$ nanoparticles (*t*_2_ = 10, 30, 60, and 240 min) were reconstructed from their tomographic analysis (Figure 1j–m). Similar to TEM and tomographic observations, the 3D structures illustrate the inflation process as shown from the green contours expanding with *t*_2_. The changes in the purple contours also indicate the expansion of the inner hollow space in an asymmetric manner, finally sitting in the middle of the nanosphere.

ET results offer quantitative analysis of structural changes during the DIAG process. The particle size of the parent APF spheres decreased from 563 ± 10 to 410 ± 10 nm of bowl‐like particles (Figure 1n; Figure S1a,b, Supporting Information) due to contraction during APF dissolution, then increased gradually with prolonged reaction time. Meanwhile, the inner hollow cavity diameter experienced little change despite of inflation along the inflated plane. The deflated and intact shell thickness (*ϕ*_d_ and *ϕ*_i_) were further quantified from tomographic analysis (Figure 1o). For S_(20, 10)_ at the initial deflation stage, *ϕ*_d_ and *ϕ*_i_ are similar at around 50 nm. Interestingly, *ϕ*_d_ decreased dramatically to less than 20 nm for S_(20, 30)_, then increased to 80 and 150 nm at *t*_2_ of 1 and 4 h. In contrast, *ϕ*_i_ increased constantly when *t*_2_ increased from 10 to 240 min. The unique concave geometry of hollow nanospheres leads to different APF polymer etching/deposition kinetics at the invaginated and intact shells as well as the wall thickness change difference (Scheme 1c). Specifically, APF polymer inside the hollow sphere can be etched into oligomers, where the invaginated shell with an inward positive curvature results in a higher oligomer solubility (*S*_p_) than that (*S*_n_) with an inward negative curvature at the intact side^[^ ^33^ ^]^ (Scheme 1c, left), initially leading to a dramatically decreased *ϕ*_d_ for S_(20, 30)_. In contrast, the APF polymer at the outer shell is more condensed and thus hardly etched, where the APF oligomers in the reaction solution tend to deposit. The oligomer deposition rate (*D*_n_) at the invaginated shell (presenting an outward negative curvature) is higher than that (*D*_p_) at the intact shell (presenting an outward positive curvature),^[^ ^33^ ^]^ thus leading to *ϕ*_d_ gradually increased for S_(20, 60)_ and S_(20, 240)_.

### 2.2 The Chemistry Origin of the DIAG Process

**Figure 2:**
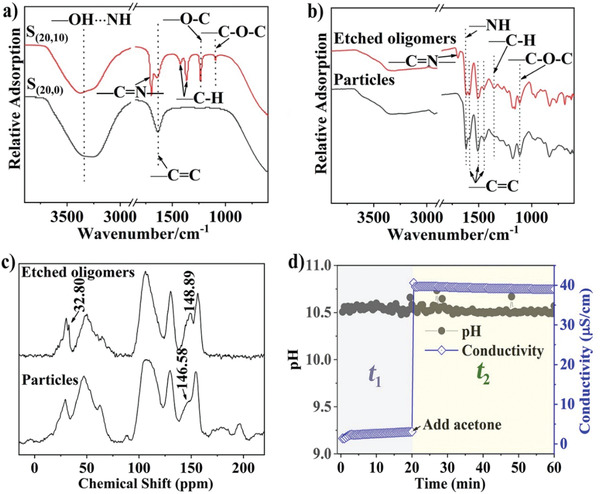
On the chemistry origin of the DIAG process. a) ATR FTIR spectra of S_(20, 0)_ and S_(20, 10)_ in solution. FTIR b) and NMR c) spectra of solid particles and etched oligomers of S_(20, 10)_. d) pH and conductivity change of the reaction solution as a function of reaction time.

Previously, acetone was considered as a solvent to dissolve polymers with low molecular weights.^[^ ^12^ ^]^ Nevertheless, the role of acetone remains to be further elucidated. In order to explore the reaction mechanism, attenuated total reflection (ATR) Fourier transform infrared (FTIR) spectra were recorded (**Figure** **2**a). For sample S_(20, 0)_ redispersed in ammonia solution to avoid the influence from residue reactants (see Figure S4 in the Supporting Information for sample preparation details), the band at 1640 cm^−1^ is assigned to the ─C═C stretching.^[^ ^34^ ^]^ After acetone treatment, bands observed at 1110 cm^−1^ can be assigned to C─O─C stretching vibrations, 1232 cm^−1^ to ─O─C stretching,^[^ ^31^ ^]^ 1364 and 1427 cm^−1^ to C─H stretching,^[^ ^34^ ^]^ all originated from APF polymers. To be noted, a new band at 1690 cm^−1^ unexpected for APF polymer is observed and assigned to ─C═N stretching.^[^ ^35^, ^36^ ^]^

To understand the origin of the ─C═N bond, the reaction solution of S_(20, 10)_ was separated by centrifugation. The etched oligomers in the supernatant were collected and freeze dried, where the ─C═N stretching (1690 cm^−1^) band was observed in FTIR spectrum, but not in the freeze dried S_(20, 10)_ particles (Figure 2b). Interestingly, when the etched oligomers in the supernatant were dried in an oven at 100 °C for 24 h instead of freeze drying, the peak of ─C═N bond was not detectable (Figure S5, Supporting Information). The above results indicate that acetone treatment has induced a chemical reaction with low crosslinked APF polymer, not just acting as a solvent. The intermediate ─C═N containing species are metastable, thus not reported before where treatment under 100 °C drying conditions was used.^[^ ^12^ ^]^

The ^13^C nuclear magnetic resonance (NMR) spectra of both freeze‐dried etched oligomers and S_(20, 10)_ particles further support the FTIR results (Figure 2c). A new peak at 32.80 ppm was observed only in the etched oligomers, which can be attributed to methyl carbon with respect to the nitrogen atom.^[^ ^37^ ^]^ The chemical shifts associated with carbon at *ortho*‐ and *para*‐positions in APF shifted from 146.6 to 148.9 ppm, presumably due to the chemical environment change after the ─C═N bond forms.^[^ ^38^ ^]^ The chemical composition of APF nanospheres before and after acetone etching did not show obvious change as evidenced in Figure S6 (Supporting Information). Compared to the etched oligomers, neither S_(20, 0)_ nor S_(20, 30)_ showed the ─C═N stretching band at 1690 cm^−1^ in the FTIR spectrum. Moreover, no peak at 32.80 ppm in the NMR spectrum was observed, suggesting that APF with a high crosslinking degree has low tendency to react with acetone.

To further understand the role of acetone in the DIAG process, the pH values and conductivity of reaction solutions were measured as a function of time at 30 s interval (Figure 2d). The time was counted from the APF polymerization catalyzed by ammonia, including both *t*_1_ and *t*_2_. The pH values showed little change during the whole process. However, the conductivity of the reaction solution was below 5 µS cm^−1^ during the polymerization stage, then increased steeply to 40 µS cm^−1^ with the addition of acetone and kept unchanged when *t*_2_ reached 60 min. For comparison, the conductivity of 3‐AP in ammonia solution without HCHO was 1 µS cm^−1^ and increased to 5 µS cm^−1^ when acetone was added (Figure S7a, Supporting Information), then experienced a slight decrease (4–5 µS cm^−1^). Similar trend was observed for HCHO in ammonia solution without 3‐AP, where the reaction solution conductivity was 1 µS cm^−1^ and increased to 6 µS cm^−1^ after acetone addition (Figure S7b, Supporting Information). The above results indicate that the addition of acetone leads to highly charged species, presumably derived from the etching of APF polymer.

Under similar synthesis condition as described above, as a control group, acetone was mixed with 3‐AP in ammonia solution in the absence of HCHO, and the mixture was tested by ATR‐FTIR after 10 min of reaction. The peak at 1690 cm^−1^ originated from the ─C═N stretching was observed, revealing acetone underwent similar chemical reaction with 3‐AP as with APF nanospheres (Figure S8, Supporting Information). These results in all support the nucleophilic addition pathway of acetone, reacting with amino/imino groups highly exposed in 3‐AP or APF with low crosslinking degree, leading to charged intermediate species during etching (Scheme 1d). However, the ─C═N species (imine) are metastable,^[^ ^39^ ^]^ the competition of HCHO induced APF repolymerization could overtake at longer reaction time, leading to more condensed APF nanostructures such as S_(20, 240)_.

The metastable nature of reactive APF oligomers with ─C═N bonds (imine) is important for understanding the structural change in the DIAG process. The key toward the unusual DIAG process lies in the addition of acetone at short *t*_1_ when the APF polymerization degree is low (e.g., 20 min), thus the polymer framework is chemically sensitive toward acetone treatment, leading to deflated hollow spheres at a short *t*_2_ of 10 min. When *t*_2_ was increased to 30 min, the reaction between acetone and low molecular weight APF oligomers is likely ongoing inside APF spheres as evidenced from the wall thickness change (Figure S1, Supporting Information; Figure 1f,g). This chemical etching process creates a high concentration of soluble oligomer with ─C═N bonds, providing a driving force for the osmosis and inflation to occur (Scheme 1b).

It should be noted that the chemical etching from nanoparticle interior is accompanied by the competition of APF repolymerization process, the latter occurs preferentially on the outer surface of the deflated nanoparticles (Scheme 1c). The inflation process is thus featured with asymmetric shell growth as evidenced in Figure 1g. It is suggested that at inner cavity where the chemical etching occurs, the solubility of the inflated surface with positive curvature is higher than that of the intact surface (*S*_p_ > *S*_n_),^[^ ^33^ ^]^ thus the invaginated shell is thinner than the intact one. At prolonged *t*_2_ when the APF repolymerization becomes dominating, the oligomer deposition rate onto the outer shell with a negative curvature is higher than that with a positive curvature (*D*_n_ > *D*_p_),^[33]^ thus the difference in wall thickness is reduced at longer *t*_2_.

### 2.3 Verification of the DIAG Process

**Figure 3:**
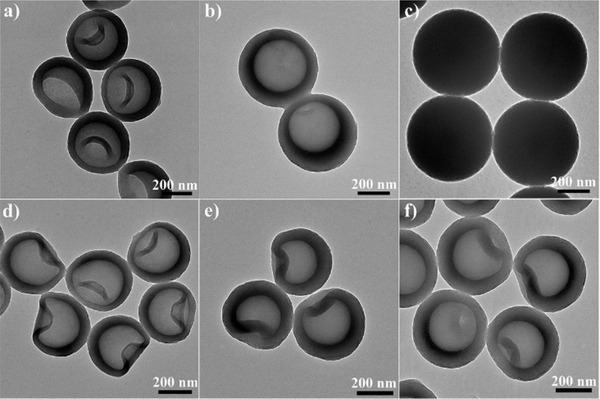
TEM images of a) S_(40, 10)_, b) S_(180, 10)_, c) S_(1440, 10)_, d) S_(120, 10)_, e) S_(120, 240)_, and f) S_(120, 1440)_.

The DIAG mechanism can be used to explain the impact of other synthesis parameters on the final structures. When *t*_1_ was prolonged (40, 180, and 1440 min) while *t*_2_ kept at 10 min, the APF polymer inside the nanosphere yielded with increasing crosslinking degrees, rendering the particles hard to be etched by acetone. Thus, no complete deflation was observed, only concave hollow spheres, intact hollow spheres with asymmetric cavity and solid sphere were obtained (**Figure** **3**a–c), respectively. By increasing the volume ratios of acetone/water, enlarged hollow cavity and degree of concavity was observed due to the fast reaction between low crosslinked APF polymer with increasing amount of acetone (Figure S9, Supporting Information). While fully deflated nanobowls were obtained with 30 mL of acetone added (Figure 1b, *V*_water_:*V*_acetone_ = 1), the particles were broken when the acetone amount increased to 31 mL (Figure S9d, Supporting Information).

When *t*_1_ was prolonged to 30 min, complete deflation of the hollow sphere cannot be observed at *t*_2_ of 10 min due to the relatively thick shell, while the shallow concavity led to quick inflation of the nanoparticles back to intact hollow spheres at *t*_2_ of 60 min (Figure S10, Supporting Information). When *t*_1_ was further increased to 120 min yielding a highly condensed shell for APF nanospheres, the inflation of concave particles to symmetric hollow spheres cannot be achieved even at increased *t*_2_ of 1440 min (Figures 3d–f). It is suggested that the DIAG process mainly applies to APF spheres with a low polymerization degree. However, when the APF nanospheres were fabricated at *t*_1_ of only 10 min, the crosslinking degree of the entire nanoparticle was too low to resist acetone etching, thus turning the milky nanoparticle suspension into a clear solution after acetone addition (Figure S11a, Supporting Information). TEM image of S_(10, 0)_ showed solid APF nanospheres at size around 400 nm (Figure S11b, Supporting Information), while not any particles can be detected in the clear solution after adding acetone (data not shown). Interestingly, the reaction solution became cloudy 10 min after the acetone addition (Figure S11a, Supporting Information), and hollow spheres with a size of around 180 nm were observed (Figure S11c, Supporting Information), possibly due to the repolymerization of APF oligomers into spheres which undergo a similar etching process. It is clear that asymmetric APF spheres with adjustable structures such as shell thickness, shell asymmetry, the degree of concavity can be controlled by varying reaction/etching time and the volume ratios of acetone/water.

### 2.4 Interface Catalysis of Concave Hollow Particles

**Figure 4:**
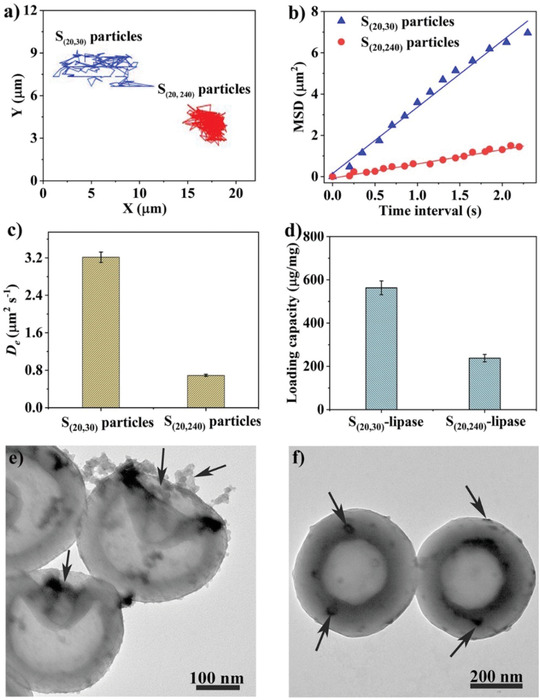
Morphological impact on particle movement and enzyme immobilization. a) Representative trajectories of S_(20, 30)_ and S_(20, 240)_ and b) corresponding mean square displacements (MSD; *n* ≥ 20). c) Effective diffusion coefficients (*D*_e_) obtained by analyzing the MSD for these two particles. d) Lipase loading capacity for S_(20, 30)_ and S_(20, 240)_, and lipase distribution analysis on e) concave and f) intact hollow particles by negative stain‐TEM.

The engineered concave architecture brings intriguing properties to these hollow particles. Compared to an intact spherical morphology, a shape of sphere‐in‐half will decrease the particle drag coefficient from 0.49 to 0.42,^[^ ^40^ ^]^ thus facilitating a faster diffusion rate. This theory was demonstrated here in hollow particles at nanoscale dimensions. Concave hollow spheres of S_(20, 30)_ and intact hollow spheres of S_(20, 240)_ were labeled with green fluorescent probe (fluorescein isothiocyanate), and their movements in water were then tracked under confocal laser scanning microscopy with representative trajectories shown in **Figure** **4**a and Videos S1 and S2 in the Supporting Information. The mean squared displacement (MSD, Figure 4b) calculated from the tracked trajectories increased linearly with time, indicating a faster displacement by concave particles of S_(20, 30)_ than spherical ones of S_(20, 240)_. The effective diffusion coefficient (*D*_e_) is thus calculated from the MSD, showing the *D*_e_ value of S_(20, 30)_ particles as 3.21 ± 0.11 µm^2^ s^−1^, which is considerably higher than that of S_(20, 240)_ particles (0.69 ± 0.02 µm^2^ s^−1^, Figure 4c).

Besides, the unique concave topology of S_(20, 30)_ particles provides an open‐pore structure which is favorable to accommodate large biomolecules.^[^ ^41^ ^]^ Here, a model enzyme of lipase was immobilized on both concave and intact hollow spheres. As shown in Figure 4d, the lipase loading capacity on S_(20, 30)_ particles was 564 ± 11 µg mg^−1^, which is more than two times of that on S_(20, 240)_ particles (238 ± 6 µg mg^−1^). Originating from the same APF nanospheres, both S_(20, 30)_ and S_(20, 240)_ particles exhibit similar specific surface area of around 18 m^2^ g^−1^ as calculated through nitrogen sorption analysis (Figure S12, Supporting Information) It is speculated that the concave shape of S_(20, 30)_ particles provides a “pocket” to load more lipase than spherical particles. This hypothesis is supported by tacking the lipase distribution on nanoparticles under TEM through negative staining. Specifically, lipase molecules that loaded on nanoparticles were stained with uranyl acetate, showing high contrast to be distinguished under electron microscopy as shown in Figure 4e,f (stained lipase marked in arrows). Interestingly, a relatively higher amount of lipase was accumulated inside or around the concave side (indicated by arrows in Figure 4e). In contrast, the intact hollow spheres of S_(20, 240)_ show an unbiased distribution across the spherical surface (Figure 4f). To be noted, the concave structure dimension plays an important role in confining the lipase inside the “pocket” of concave nanoparticles. As shown in Figure S13 (Supporting Information), the nanobowls of S_(20, 10)_ with a semispherical concave structure and diameters of more than 200 nm showed a lipase loading capacity of only 313 ± 12 µg mg^−1^, which is around half of that for S_(20, 30)_. S_(20, 60)_ having a relatively shallow concavity presented a similar lipase loading amount compared to S_(20, 30)_. The results suggest that the concave structure with an opening pore dimension less than 100 nm would benefit the lipase molecules accumulation, thus leading to high loading capacity for both S_(20, 30)_ and S_(20, 60)_.

**Figure 5:**
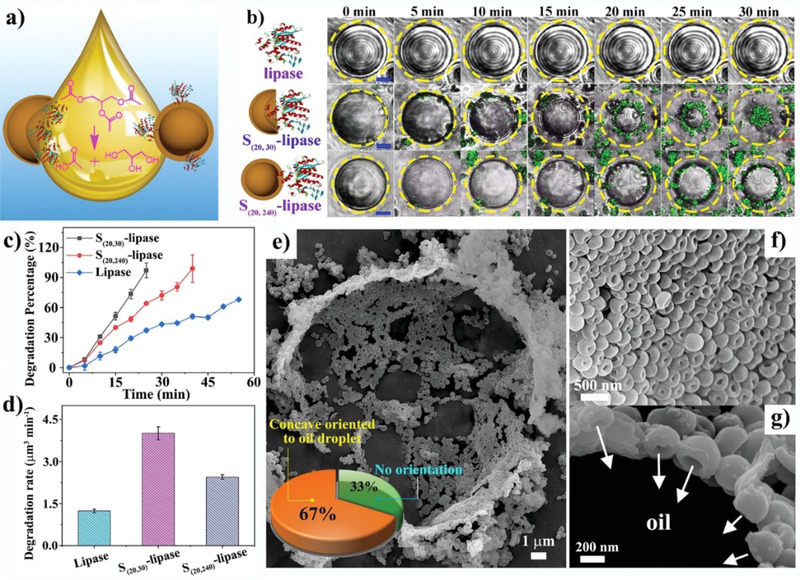
Lipase immobilized nanoparticles for interface catalytic degradation of tributyrin droplets. a) Schematic illustration of the nanoparticle interface assembly and tributyrin breakdown via concave and intact hollow spheres. b) Optical microscopy of tributyrin droplets degradation processes by lipase and lipase conjugated nanoparticles (scale bar is 5 µm). c) Degradation kinetics of tributyrin droplets calculated from their volume change. d) Degradation rates of oil droplet calculated through linear fitting of corresponding kinetics. e) SEM image of S_(20, 30)_‐lipase particles assembled at the oil droplet surface. Representative SEM images in high magnification viewed from f) inside of oil droplet and g) side edge, with nanoparticle orientations analyzed by counting 3 regions of more than 50 particles in SEM images (e, inset).

The rapid mobility, high and asymmetric lipase loading of concave hollow spheres make these particles promising nanocatalysts to be diffused at the oil–water interfaces to promote enzymatic oil degradation. As shown in **Figure** **5**a, conventional lipase‐conjugated nanoparticles in spherical morphology can only expose part of their enzyme toward the inner oil core when assembled at the biphasic interface. In contrast, concave hollow spheres with lipase accumulated at one side of the particle are expected to have most of the enzyme facing toward the oil phase due to the relatively hydrophobic nature of protein domains, beneficial for the formation of Pickering emulsion droplets. This favorable assembly orientation of concave spheres at oil–water interface would enhance their enzymatic catalysis efficiency, thus leading to fast oil degradation. Moreover, fast motility of concave spheres can also shorten their diffusion time before reaching the oil droplet,^[^ ^42^ ^]^ or behaving as more effective catalysts than intact spheres when loaded with enzymes.

To support our hypothesis, tributyrin was introduced in water forming oil‐in‐water emulsion. Both concave and intact hollow spheres of S_(20, 30)_ and S_(20, 240)_, labeled with FITC and loaded with lipase, were added into the emulsion at a fixed distance of 1 cm far from oil droplets under field of view. It is expected that the lipase transported via these nanoparticles toward oil–water interfaces leads to the breakdown of tributyrin into water‐soluble butyric acid and glycerol. The dynamic light scattering (DLS) analysis of lipase loaded concave nanoparticle (S_(20, 30)_‐lipase) and intact hollow spheres (S_(20, 240)_‐lipase) showed their particle size of 464 and 513 nm, respectively, with the polydispersity index (PDI) both being around 0.2, indicating their well dispersity in aqueous solutions. As shown in Figure 4b, representative oil droplets (initial size of ≈20 µm) treated by S_(20, 30)_‐lipase, S_(20, 240)_‐lipase and free lipase were captured under confocal microscopy as a function of time (merged with green fluorescent and bright‐field images). No obvious size decay was observed for oil droplets treated by free lipase (45 µg mL^−1^) within 30 min. In contrast, the oil droplet size decreased dramatically over time treated with S_(20, 30)_‐lipase at the same enzyme concentration, ending with full degradation at 30 min of incubation. Compared to the concave spheres, lipase‐loaded intact hollow spheres (S_(20, 240)_‐lipase, dosed at equivalent lipase concentration) showed a slower kinetics of tributyrin degradation than S_(20, 30)_‐lipase, with oil droplet remained around 10 µm in diameter over 30 min of treatment. By calculating the droplet volume change, the oil degradation kinetics was presented in Figure 5c. Almost linear degradation of the oil droplets was observed with concave spheres having the highest rate of 4.03 µm^3^ min^−1^, which is more than 3 times than that of free lipase and around 2 times of intact hollow spheres (Figure 5d). The oil degradation kinetics of S_(20, 240)_ and S_(20, 30)_ was also compared at the same nanoparticle concentration (80 µg mL^−1^). As shown in Figure S15 (Supporting Information), the intact hollow spheres showed a significantly slower oil degradation rate of only 1.97 µm^3^ min^−1^ than concave nanoparticles (4.03 µm^3^ min^−1^).

The diffusion of these fluorescently labelled nanoparticles toward the water–oil interface was observed via confocal microscopy (Figure 5b), where concave spheres quickly gathered and covered the oil droplets, facilitating fast catalytic degradation. To directly observe the assembly behavior of concave spheres at the biphasic interface, emulsion treated with S_(20, 30)_‐lipase was lyophilized for SEM analysis. As shown in Figure 5e, solvent sublimation left a partially collapsed spherical shape with a diameter of about 19 µm, similar to the size of tributyrin droplet. The orientations of these concave hollow spheres assembled at the water–oil interface were further revealed via SEM at higher magnifications (Figure 5f,g). Viewing from inside of the droplet, more than 67 ± 1% of the particles exposed their concave faces toward the oil core (Figure 5f, inset). Side view of the nanoparticle assembly further demonstrates this preferred orientation of concave spheres probed at biphasic interfaces (Figure 5g). Interestingly, S_(20, 30)_ particles without lipase conjugation can also pack at the emulsion surface but without an obvious orientation preference (Figure S16a, Supporting Information). Compared to S_(20, 30)_‐lipase which exhibited 67 ± 1% of nanoparticles exposing their concave face toward oil, bare S_(20, 30)_ nanoparticles without lipase showed a significantly lower percentage of nanoparticles (38 ± 1%) presenting their concave side to the oil droplet (Figure S16b, Supporting Information). The unique concave architecture of hollow spheres mediates the asymmetric loading of enzyme, which in turn directs the assembly of these particles at the oil–water interfaces exposing most lipase to the oil for efficient interface catalysis.

## 3 Conclusion

In summary, an intriguing DIAG approach is developed to fabricate hollow particles with intricate concave nanostructures, and acetone is redefined as involved in the nucleophilic addition reaction of APF polymers for etching. With the DIAG approach, hollow nanosphere can be easily tailored from deflation to inflation consecutively by playing with the APF etching and deposition kinetics, offering unique concave nanostructures with asymmetric wall thickness that conventional synthesis approaches cannot achieve. The asymmetric nature of these concave particles results in localized enzyme immobilization and oriented assembly at the biphasic interface for enhanced enzymatic catalysis. Our findings provide a platform for custom‐designed concave hollow nanostructures, and pave the way for rational synthesis of more polymeric capsules with intriguing properties.

## Conflict of Interest

The authors declare no conflict of interest.

## Supporting information

Supporting Information

Supporting Video S1

Supporting Video S2
