# Supplementary material for: Shaping Nanoparticles for Interface Catalysis: Concave Hollow Spheres via Deflation–Inflation Asymmetric Growth
Source: Adv Sci (Weinh). 2020 May 19;7(13):2000393. doi: 10.1002/advs.202000393 (PMC7341089; doi:10.1002/advs.202000393)
Supplement: Supplementary file 1 — Supporting Information [file ADVS-7-2000393-s001.pdf]

Copyright WILEY-VCH Verlag GmbH & Co. KGaA, 69469 Weinheim, Germany, 2018.

Supporting Information

Shaping Nanoparticles for Interface Catalysis: Concave Hollow Spheres via Deflation-Inflation Asymmetric Growth

Rongtai Yu[1,2+], Xiaodan Huang[2+], Yang Liu[2,3], Yueqi Kong[2], Zhengying Gu[2,3], Yang Yang[2], Yue Wang[2], Wenhuang Ban[2], Hao Song[2], Chengzhong Yu[2,3]**

[+] These authors contribute equally to this work.

1 Dr. R. Yu
School of Materials Science and Engineering
Jingdezhen Ceramic Institute
Jingdezhen, 333403, Jiangxi, P. R. China

2 Dr. R. Yu, Dr. X. Huang, Dr. Y. Liu, Y. Kong, Z. Gu, Y. Yang, Y. Wang, W. Ban, Dr. H. Song, Prof. C. Yu
Australian Institute for Bioengineering and Nanotechnology
The University of Queensland
Brisbane, Queensland 4072, Australia
Email: h.song6@uq.edu.au; c.yu@uq.edu.au

3 Dr. Y. Liu, Z. Gu, Prof. C. Yu
School of Chemistry and Molecular Engineering
East China Normal University
Shanghai 200241, P. R. China

Experimental:

Chemicals. 3-aminophenol (3-AP, 98%), formaldehyde solution (37 wt%), ammonia aqueous solution (NH_4OH, 25 wt%), acetone (99 wt%), ethanol (99%), tributyrin (97%), Fluorescein 5(6) isothiocyanate dye (FITC, 90%), lipase (from Candida Rugosa, 706 unit/mg solid), glutaraldehyde (GA, 25% in water) were purchased from Sigma-Aldrich. All chemicals were used as received without further purification.

Synthesis of Concave Asymmetry Hollow Nanospheres. Typically, 0.1 g 3-AP was mixed with deionized water (30 mL) at room temperature. Then formaldehyde solution (0.1 mL) and ammonia (0.05 mL) were added under stirring of around 600 rpm after 3-AP was dissolved completely. After 20 min of reaction, 30 mL of acetone were added to selectively remove the low cross-linked APF polymer inside nanospheres. At a certain reaction time, the as-synthesized hollow particles were collected by centrifugation at 15,000 rpm for 5 min, and then washed with deionized water, followed by freeze-drying of the particles yielding final products. Nanoparticles synthesised without stirring and centrifugation were fabricated by following the above synthesis procedure except keeping the reaction solution static, and obtained by direct lyophilisation of the nanoparticles for following analysis.

Grafting of Fluorescein 5(6) isothiocyanate dye (FITC). 30 mg $S_{(20,t2)}$ particles were suspended in 20 mL of ethanol, followed by sonication for 10 min. Then, 5 mg of FITC was added and stirred for 4 hour under dark. FITC tagged particles were collected by centrifugation followed by 3 times of washing with ethanol to remove the free dye.

Lipase immobilization on nanoparticles. 2 mg of $S_{(20,\ t2)}$ particles labelled with FITC were suspended in 900 µL of phosphate buffer saline (PBS) and sonicated for 10 min. Then, 100 µL glutaraldehyde (0.25 w%) was added and vortexed with particles for 30 s. The mixture was shaken for 3 h at 200 rpm at room temperature. Afterwards, the particles were collected by centrifugation and washed with PBS for 3 times. To enable enzyme immobilization, these nanoparticles were re-suspended in 500 µL of PBS solution, mixing with another 500 µL of lipase solution (4 mg/L), then shake at 200 rpm under 10 ºC for 12 h. Finally, the lipase-immobilized nanoparticles were obtained by centrifugation followed with 3 times of washing using PBS, and then re-suspended in 1 mL PBS solution for following use.

Lipase loading capacity quantification. By immobilizing lipase on hollow nanoparticles as described above, the supernatants after lipase loading process were collected for protein concentration

quantification by utraviolet spectrophotometer (UV) analysis. Standard curve regarding the absorbance of lipase solution at a serials of concentrations were measured and plotted for supernatant concentration quantification.

Preparation of the tributyrin-water emulsion. 20 µL of tributyrin was added into 500 µL PBS solution, then mixed by strong hand-shaking for 1 min to form uniform emulsion and used for following tests immediately.

Optical microscopy analysis of nanoparticle movement. Lecia SP8 confocal microscope was used to observe and record images/videos for the nanoparticle movement. The FITC-labelled nanoparticle samples were suspended into PBS, with the fluorescent signal captured and videoed for 15 s with a frame rate of 50 fps. Video and mean square displacements (MSD) were analysed by image J and Matlab 2019 a.

Oil degradation observation and calculation. To analyse the oil degradation behaviour assisted by these lipase-loaded nanoparticles, PBS solution with 20 µL tributyrin were first shaken for 1 min to form uniform emulsion. Then the emulsion was immediately pour into a petri dish for microscopy analysis using Lecia SP8 confocal microscope. Lipase-loaded, FITC-labelled hollow nanoparticles were then injected into the emulsion solution with a fixed distance of 1 cm to the oil droplets under microscope field of view. The diameter of the oil droplet from the optical images by Leica SP8 confocal microscope were measured at a function of time, and the nanoparticle diffusion behaviour were analysed by identifying the green fluorescence. The oil degradation percentage were calculated according to the volume change of the oil droplet:

$$Degradation\% = [(D^3_{before} - D^3_{after}) / D^3_{before}] \times 100\%$$

Where D_{before} is the original oil droplet diameter, D_{after} is the droplet diameter after treatment.

General Characterization. Scanning electron microscopy (SEM) were carried out using a JEOL JEM 7800F at 1 kV. Transmission electron microscopy (TEM) images were captured by using JEM-2100 microscope operated at 200 kV. Nitrogen sorption analysis was conducted using Micromeritics Tristar 3020 under liquid nitrogen temperature, before analysis samples were degassed under 150 °C for 6 h. Fourier transform infrared (FTIR) spectra were obtained using Nicolet 6700. Electron tomography (ET) specimens were investigated by a FEI Tecnai F30 operated at 300 kV with bright-field TEM mode. Nuclear magnetic resonance (NMR) were carried on a solid state Bruker ADVANCE III spectrometer. Optical microscope were tested by Lecia SP8 confocal microscope.

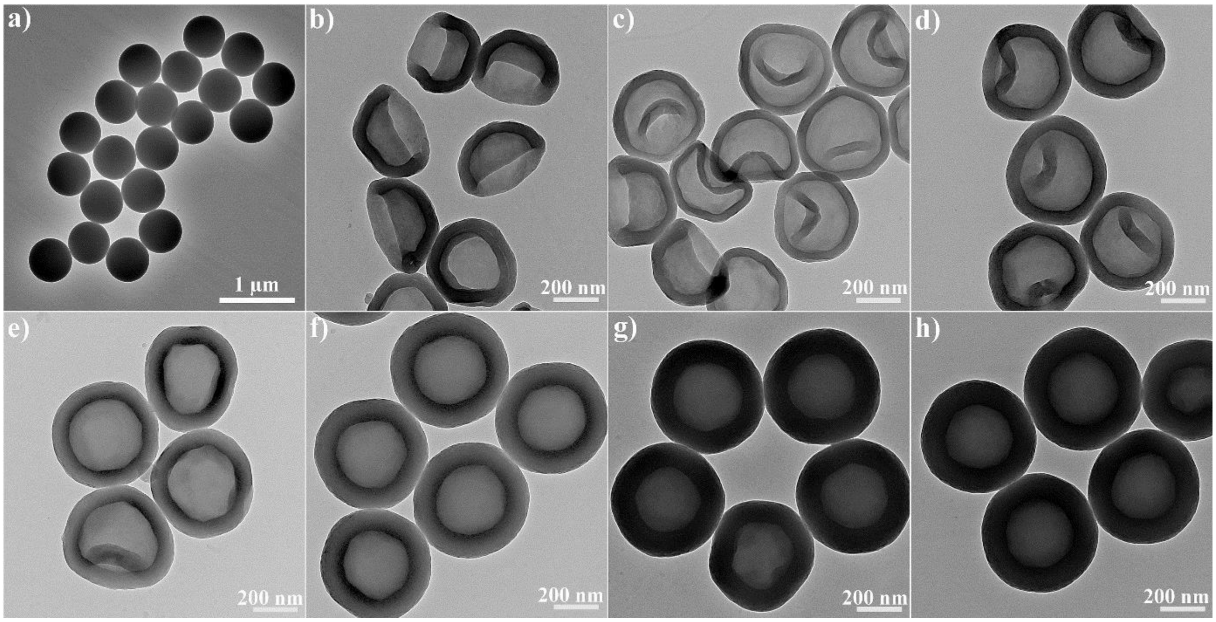

Figure S1 Characterization of the structure evolution during DIAG process. TEM image of APF nanospheres of $S_{(20, 0)}$, $S_{(20, 10)}$, $S_{(20, 30)}$, $S_{(20, 60)}$, $S_{(20, 120)}$, $S_{(20, 240)}$, $S_{(20, 360)}$ and $S_{(20, 540)}$, respectively.

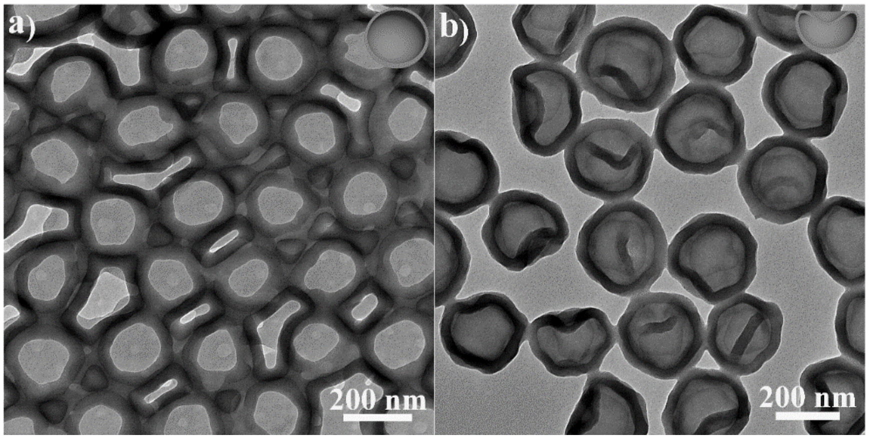

Figure S2. TEM images of (a) $S_{(20,1)}$ and (b) $S_{(20, 5)}$

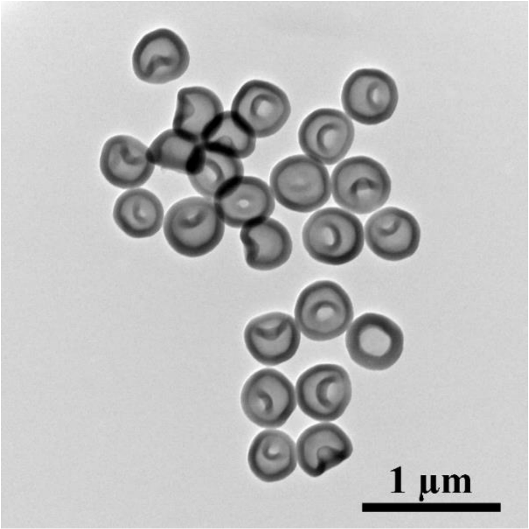

Figure S3. TEM image of as-synthesized $S_{(20,30)}$ obtained at synthesis condition without stirring and centrifugation.

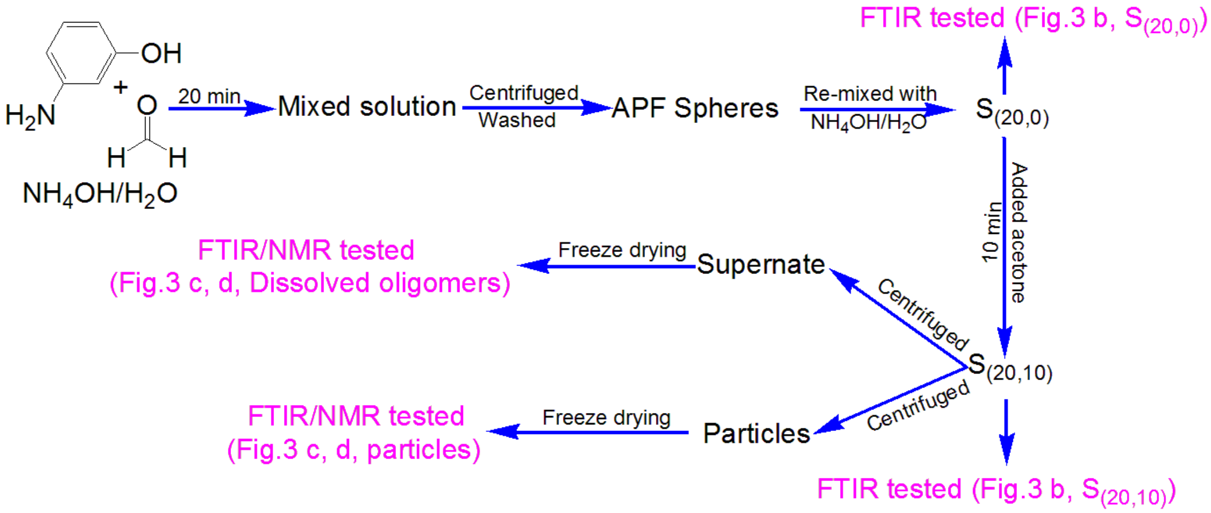

Figure S4 The sample preparation process for FTIR analysis.

(Note: To exclude the interference of residual 3-AP and oligomers in the polymerization reaction solution on the FTIR characterization, the APF nanospheres were centrifuged and washed before re-dispersed in the ammonia/water solution for further processing and analysis. The morphology of the particles processed in this case maintained the same as described under normal synthesis process.)

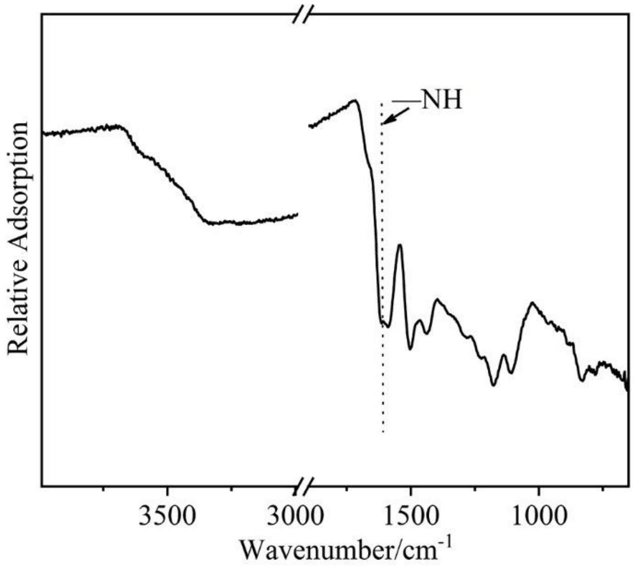

Figure S5 FTIR spectrum of etched oligomers collected from the supernatant of S(20,10) and dried in an oven at 100 ºC for 24 h.

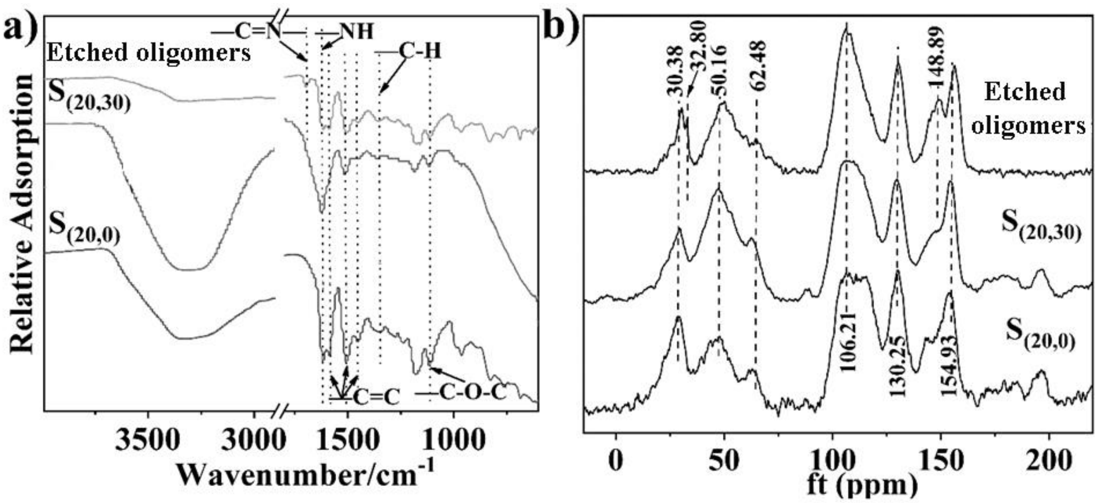

Figure S6. (a) ATR FTIR spectra and (b) NMR spectra of S(20, 0) and S(20, 30) compared to the etched oligomers.

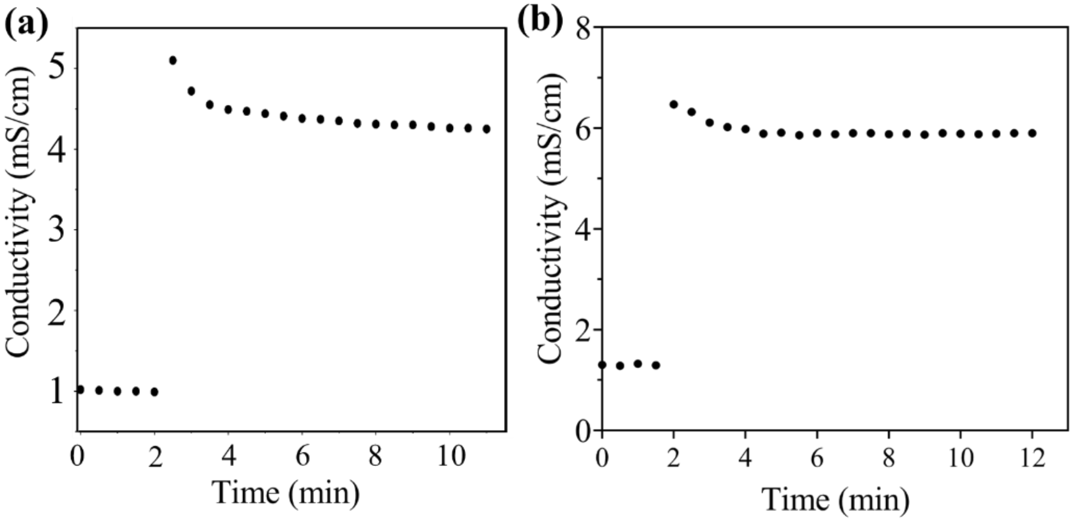

Figure S7. Conductivity of (a) 3-AP (0.1 g in 30 mL water) and (b) formaldehyde (0.1 mL in 30 mL water) reacted with acetone (30 mL).

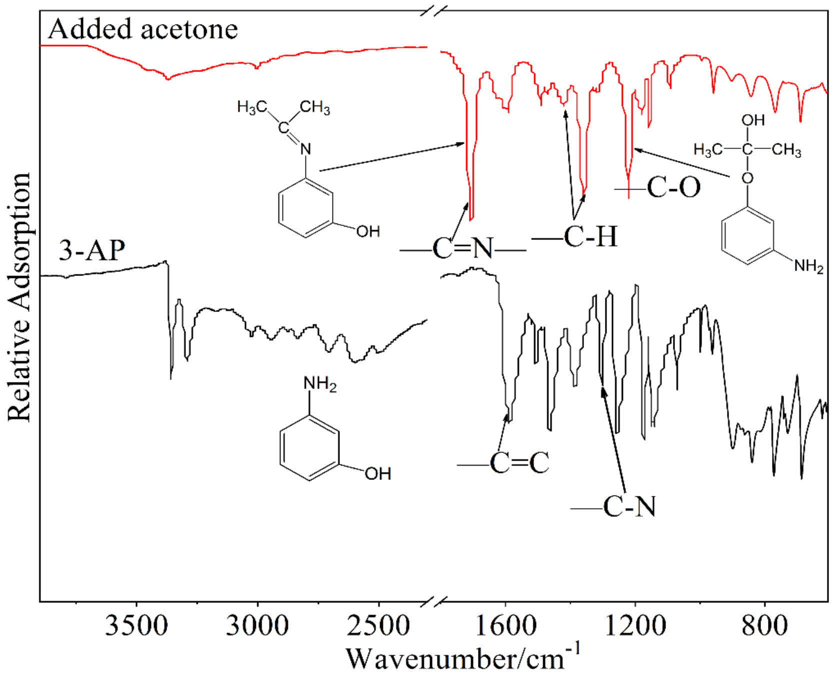

Figure S8 FTIR analysis of 3-AP (0.1 g in 30 mL water) and 3-AP reacted with acetone (3-AP in 30 mL water by adding 30 mL acetone).

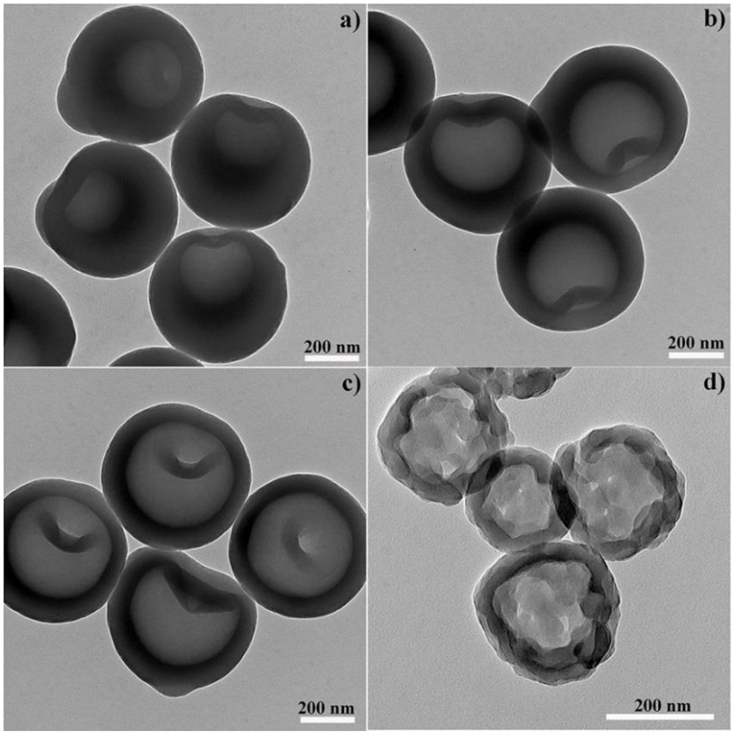

Figure S9 TEM images of APF nanospheres (t_1=20 min, t_2=10 min) reacted with different amount of acetone. (a) 15 mL; (b) 20 mL; (c) 25 mL; (d) 31 mL.

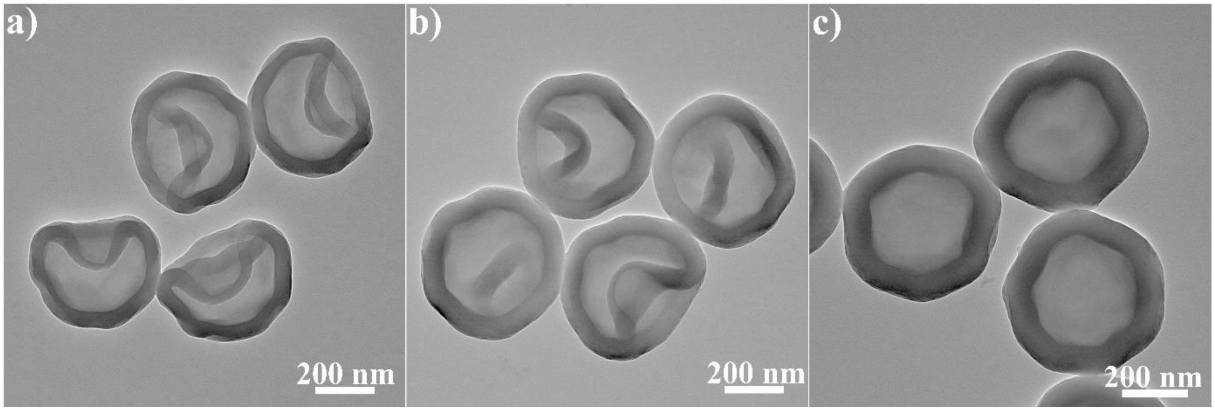

Figure S10 TEM images of (a) $S_{(30, 10)}$, (b) $S_{(30, 30)}$, (c) $S_{(30, 60)}$.

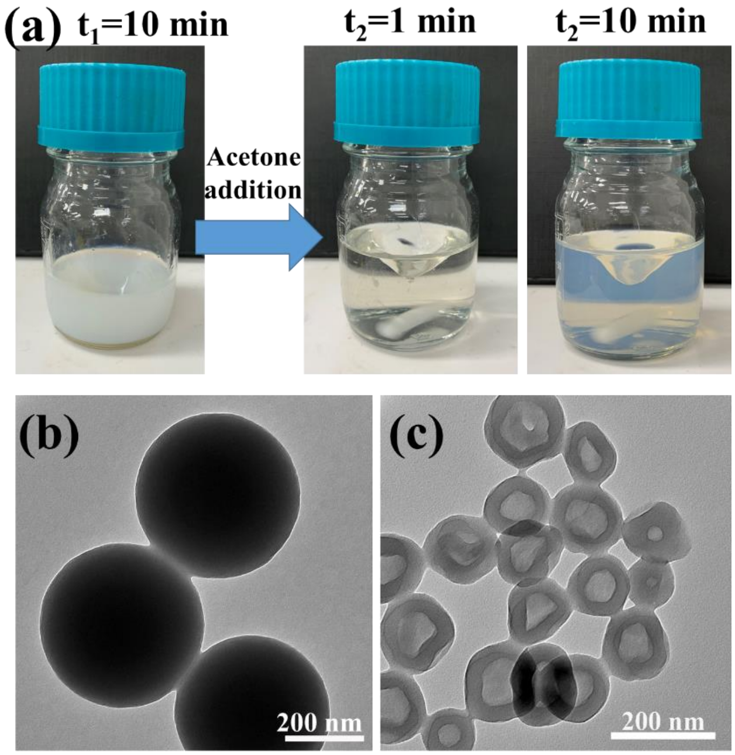

Figure S11. (a) Photographs of APF nanospheres synthesized at t_1=10 min, followed by 30 mL of acetone addition, and TEM images for (b) $S_{(10,0)}$ and (c) $S_{(10,10)}$.

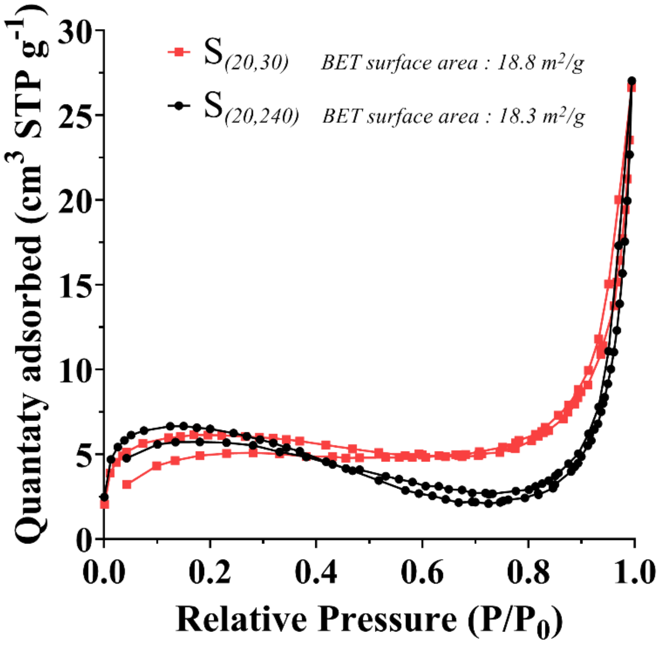

Figure S12. Nitrogen sorption isotherms of APF concave particles of $S_{(20, 30)}$ and hollow particles of $S_{(20, 240)}$.

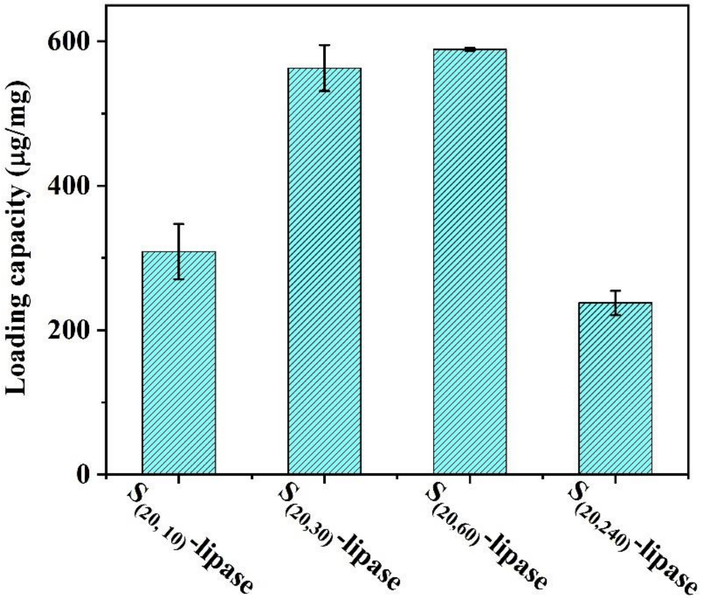

Figure S13. Lipase loading capacity for $S_{(20,10)}$, $S_{(20,30)}$, $S_{(20,60)}$ and $S_{(20,240)}$

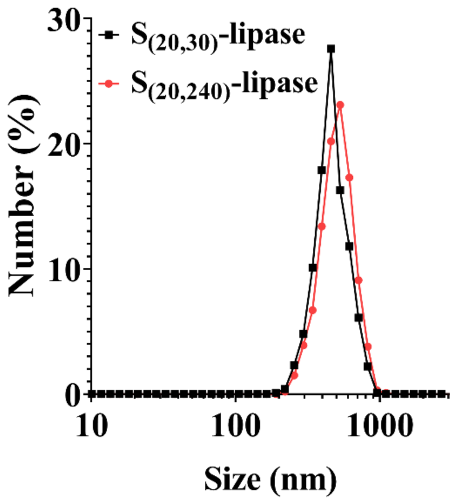

Figure S14. DLS analysis of $S_{(20,30)}$-lipase and $S_{(20,240)}$-lipase in water.

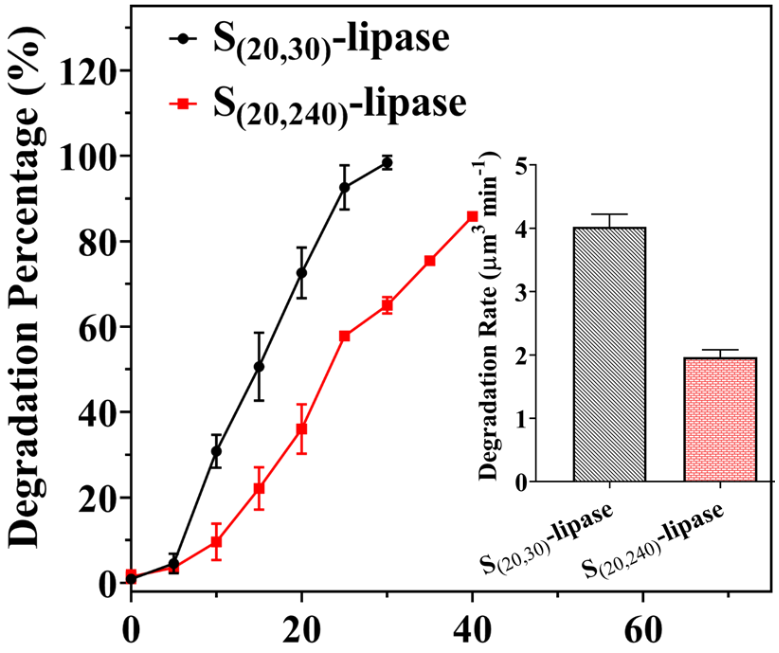

Figure S15. Degradation kinetics of tributyrin droplets by lipase loaded concave and intact hollow spheres calculated from their volume change. Inset shows the degradation rates of oil droplet calculated through linear fitting of corresponding kinetics.

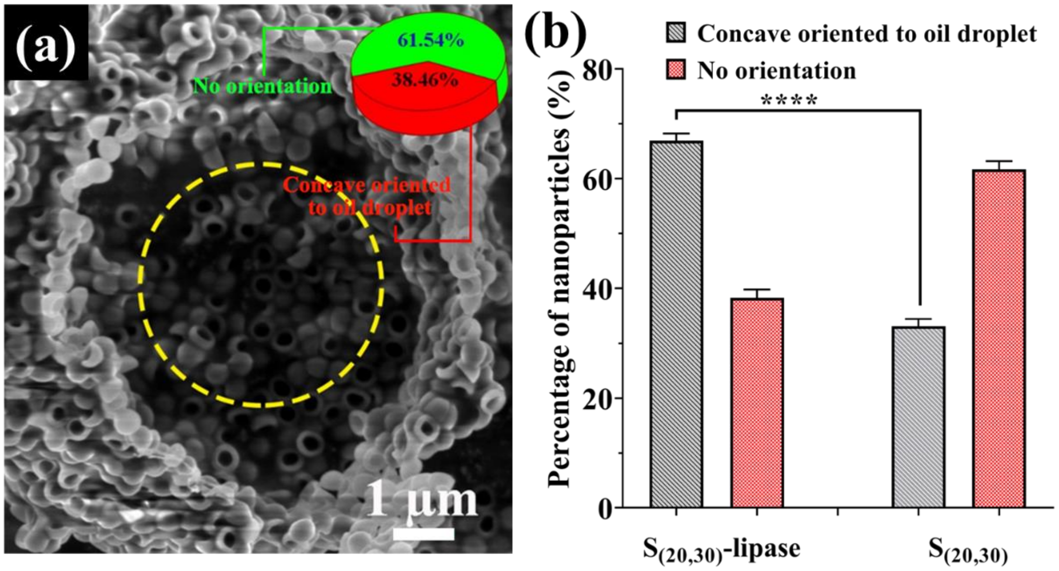

Figure S16 (a) Typical SEM image of concave nanoparticles of $S_{(20, 30)}$ (without lipase immobilization) assembled at the oil droplet surface after freeze-drying, and statistically analysis of the particle orientations for concave nanoparticles with and without lipase conjugation. Data are collected by counting 3 typical regions with more than 50 particles and statistically analysed using t-test, with ****P < 0.0001.

Video S1, Representative $S_{(20, 30)}$ particles with tracked trajectory

Video S2, Representative $S_{(20, 240)}$ particles with tracked trajectory
